# COVID-Well: Evaluation of the Implementation of Supported Wellbeing Centres for Hospital Employees during the COVID-19 Pandemic

**DOI:** 10.3390/ijerph17249401

**Published:** 2020-12-15

**Authors:** Holly Blake, Mehmet Yildirim, Ben Wood, Steph Knowles, Helen Mancini, Emma Coyne, Joanne Cooper

**Affiliations:** 1 School of Health Sciences, University of Nottingham, Nottingham NG7 2HA, UK; ntxmy4@nottingham.ac.uk; 2 NIHR Nottingham Biomedical Research Centre, Nottingham NG7 2UH, UK; 3 Human Resources, Nottingham University Hospitals NHS Trust, Nottingham NG7 2UH, UK; ben.wood@nuh.nhs.uk (B.W.); steph.knowles@nuh.nhs.uk (S.K.); helen.mancini@nuh.nhs.uk (H.M.); 4 Clinical Psychology Department, Nottingham University Hospitals NHS Trust, Nottingham NG7 2UH, UK; emma.coyne@nuh.nhs.uk; 5 Nursing and Midwifery, Nottingham University Hospitals NHS Trust, Nottingham NG7 2UH, UK; joanne.cooper3@nuh.nhs.uk

**Keywords:** COVID-19, pandemic, psychological wellbeing, mental health, wobble rooms, wellbeing centres

## Abstract

Supported Wellbeing Centres have been set up in UK hospital trusts in an effort to mitigate the psychological impact of COVID-19 on healthcare workers, although the extent to which these are utilised and the barriers and facilitators to access are not known. The aim of the study was to determine facility usage and gather insight into employee wellbeing and the views of employees towards this provision. The study included (i) 17-week service use monitoring, (ii) employee online survey with measures of wellbeing, job stressfulness, presenteeism, turnover intentions, job satisfaction, and work engagement, as well as barriers and facilitators to accessing the Wellbeing Centres. Over 17 weeks, 14,934 facility visits were recorded across two sites (peak attendance in single week *n* = 2605). Facilities were highly valued, but the service model was resource intensive with 134 wellbeing buddies supporting the centres in pairs. 819 hospital employees completed an online survey (88% female; 37.7% working in COVID-19 high risk areas; 52.4% frontline workers; 55.2% had accessed a wellbeing centre). There was moderate-to-high job stress (62.9%), low wellbeing (26.1%), presenteeism (68%), and intentions to leave (31.6%). Wellbeing was higher in those that accessed a wellbeing centre. Work engagement and job satisfaction were high. Healthcare organisations are urged to mobilise access to high-quality rest spaces and psychological first aid, but this should be localised and diversified. Strategies to address presenteeism and staff retention should be prioritised, and the high dedication of healthcare workers should be recognised.

## 1. Introduction

### 1.1. Psychological Wellbeing in Healthcare Workers

It is well accepted that working in healthcare can be emotionally and physically demanding. The health and wellbeing of healthcare workers is associated with patient safety and experience, staff retention, and economic burden to the NHS from sickness absence [1]. Forward-thinking NHS trusts therefore invest routinely in staff health and wellbeing, and this reaps benefits in terms of improvements in health behaviours, reductions in sickness absence, and improvements in job satisfaction and organisational commitment [2]. The novel coronavirus (SARS-CoV-2) commonly presents as a severe acute respiratory disease referred to as coronavirus disease-2019 (COVID-19). In March 2020, the COVID-19 outbreak in the UK was declared a pandemic. It was widely recognised that there was (and remains) an urgent need to support the psychological wellbeing of the healthcare workforce at this time [3]. Preliminary findings from the ICON study, a national survey of nurses and midwives, found that one third of nurses and midwives were experiencing ‘severe’ mental health issues during the first wave of the pandemic in the UK [4,5], reporting concerns over the risks to their health and that of their families, as well as inadequacy of training and lack of access to personal protective equipment (PPE).

The COVID-19 pandemic has generated an increased sense of urgency to mobilise crisis-response provisions to protect the wellbeing of healthcare workers.

### 1.2. The Importance of Time-Out and Work Breaks

COVID-19 mental wellbeing interventions have been developed with some agility. A digital support package was developed within three weeks of the declaration of a pandemic in the UK, to promote psychological wellbeing in health and care workers [6,7]. This contains evidence-based guidance and signposting around mental health, creation of psychological safe workplaces, leadership and communication during COVID-19, and self-care. Within this package, the importance of self-care, and time-out for rest and work breaks is advocated. Time-out is increasingly important for healthcare workers, particularly those on the frontline who are more exposed to high levels of stress and burden from the pandemic. The concept of time-out in the workplace contrasts significantly with its original use. In the first half of the 20th century, time-out was used primarily as a means of behavioural control with inmates in secure environments and patients with mental health disorders [8]. In this context, time-out refers to an opportunity for respite, providing down time for workers to rest and re-charge. Time-out is already advocated in various forms and different contexts. For example, compulsory coffee breaks have been advocated for primary care general practitioners as a way of preventing burnout [9]. The positive effects of quiet time on hospital wards has been observed. In clinical areas, the implementation of ‘quiet time’ providing 1 h of rest during which only admissions, essential tests, discharges and procedures are permitted, reduced noise levels on wards, provided opportunities for teams to take time out together, and increased happiness in staff [10]. In operating theatres, systematic time-out is used to prevent ‘wrong-site’ surgeries, improve patient safety and quality of care [11,12]. The need for time-out is amplified in high stress situations, and adequate breaks are associated with reduced morbidity in healthcare staff working with patients in an outbreak of any emerging virus in clinical settings [13].

### 1.3. Dedicated Wellbeing Centres

Many hospital trusts have engaged teams to rapidly create respite spaces for staff [14,15], albeit the same concept has been labelled in many ways: ‘wobble rooms’, ‘time-out rooms’, ‘chill-out rooms’ ‘safe rooms’, ‘rainbow rooms’ and ‘wellbeing centres’.

These facilities are usually located in non-COVID areas and for frontline workers they provide an opportunity for staff to remove themselves from the clinical environment and gain solace from the pressures of dealing with coronavirus. They are intended to provide an optimistic and positive atmosphere to help staff with the impact of the crisis—small enough to be perceived as ‘homely’ spaces but large enough to maintain privacy and appropriate social distancing. These rooms often provide quiet areas for rest and relaxation, somewhere to eat or drink, and for others a chance to talk with others to strengthen camaraderie and support. Staff can access pastoral support in terms of counselling or psychological support including psychological first aid, either face-to-face or by video link. Sensory items may be provided (e.g., low-level lighting, ‘stress balls’, aromatic oils and lava lamps) in addition to wellbeing resources and signposting. There are notable parallels between environments designed for staff wellbeing, and the preventative and de-escalating ‘sensory rooms’ and ‘comfort rooms’ that are already used with patients, in psychiatric inpatient environments [16,17] and to support self-management in people with chronic conditions, such as long-term pain. In addition to physical spaces, some trusts are offering virtual wobble rooms for healthcare workers, providing regular online group-based support focused on honest expression of feelings and self-care [18].

### 1.4. Study Aim

There is much to learn from the rapid implementation of these facilities aimed at supporting the psychological wellbeing of healthcare workers during and after a pandemic. An evaluation of the implementation of wellbeing centres at an acute NHS trust in England was undertaken (the COVID-Well Study) with the aim of providing insights into the delivery and impacts of this COVID-19 response, as well as providing lessons learned to help shape future provisions as we advance to the ‘new normal’. The objectives were to gather data to provide insights into (a) workforce wellbeing and job perceptions, (b) centre attendance, barriers and facilitators to usage; and (c) views of healthcare workers towards the wellbeing centres and support workers.

## 2. Methods

### 2.1. Study Design

This study included service use monitoring data, collected during a 17-week period between April and July 2020 (during the first COVID-19 peak in the United Kingdom (UK)), and an online questionnaire survey for hospital employees conducted in July and August 2020 (conducted post-peak). The research was reviewed and approved by University of Nottingham Faculty of Medicine and Health Sciences Research Ethics Committee (Ref. 16-0520).

### 2.2. Participants and Setting

Eligible participants were hospital employees from any site of an acute hospital trust in the UK. The term ‘employees’ is used in this context to refer to all paid employees as well as bank staff and contracted hospital volunteers working on the study sites during the pandemic.

### 2.3. The Intervention: Wellbeing Centres and Wellbeing Buddies

This intervention was a COVID-19 response to provide psychological support and respite to the healthcare workforce during the pandemic, e.g., [19]. Two wellbeing centres were rapidly implemented at two different sites of an acute hospital trust (Site A and Site B). The centres opened on 6 April 2020. The intention was that the centres would be relaxing spaces, and as such they had comfortable seating, relaxing music, low-level lighting, plants and an aromatherapy pod. Refreshments were available, and for a limited time period in the early days of opening there were charitable donations for employees (including personal care packages, wash bags, toiletries, snacks, and washable uniform bags). Both sites had comparable facilities, although Site A was a purpose built wellbeing room, and Site B was a converted hospital ward. Employees could use the spaces for quiet time out, social contact, or to access emotional support. The centres could be accessed by employees between 08:00 and 20:00 on seven days of the week. In each centre, there were two wellbeing support workers available at any time, called ‘wellbeing buddies’. Some, but not all, of the buddies had prior experience in counselling or patient-facing roles that involved ‘active listening’, although there were no pre-requisites for this role as all volunteers received training and support. One hundred and thirty-four buddies were trained in psychological first aid (PFA) by clinical psychologists, who also provided the buddies with regular supervision and drop-in sessions to address their queries, provide mentoring and psychological support. Psychological first aid (PFA) is described as “a humane, supportive response to a fellow human being who is suffering and who may need support” [20]. PFA involves the provision of non-intrusive psychological support, assessment of needs and concerns, and helping people to address basic needs (e.g., hydration or information). It involves listening to people without pressuring them to talk, comforting and calming people in distress, protecting people from further harm and helping people to connect to information, services and social support. PFA is the globally recommended training for supporting people during emergencies and offers guidance on delivering psychosocial care in the immediate aftermath of the emergency event (or a perceived emergency), such as dealing with a patient with COVID-19. Training in PFA is advocated by the UK government and Public Health England, for staff and volunteers who are at the forefront of the national coronavirus response.

The buddies themselves kept the rooms clean and safe, ensuring that visitors adhered to health and safety regulations, including social distancing guidelines. They provided the refreshments and engaged in conversations where it was appropriate (e.g., if the visitor sought out social contact rather than quiet time). Their primary role was to engage in ‘active listening’, allowing employees to raise particular issues, discuss personal or work-related challenges or simply engage in social conversation for time out. Their training prepared them for discussions of an emotional nature, and their role was to listen and signpost employees appropriately if the buddies identified that the employee may benefit from further support. Partitioned areas in each centre allowed the privacy and space for the buddies to provide emotional support and signposting. Signposting included provision of details about local and national supportive services, such as occupational health services, welfare support, services supporting transport, childcare and employee COVID-19 testing, as well as telephone crisis helplines for healthcare workers, employee assistance programmes, health and wellbeing apps (e.g., Headspace: mindfulness; Sleepio: managing sleep and shift work) self-care resources, and recommendations to access counselling services or a general practitioner (GP) where appropriate. Buddies were NHS employees who had reduced workload in their main roles during the pandemic due to temporary closures of clinics or services. These staff members opted into the wellbeing buddy role to provide peer-to-peer support and signposting in the centres. The minimum time commitment for any buddy was a single 4-h shift and the level of time commitment varied with some buddies completing 1–2 shifts in total, and others completing several shifts per week. However, all buddies continued to be employed in their main job while taking time out of this role to volunteer as a wellbeing buddy in the centres. Towards the end of the study period, buddies who had worked any shifts in the wellbeing centres during the pandemic were required to return fully to their usual roles. This transition coupled with analysis of usage data, informed a decision to change the centre opening hours to Monday–Friday 10:00–16:00 towards the end of the study period (week 9). The intervention leadership and delivery team had no role in the design of the research study, or the analysis and interpretation of data.

### 2.4. Procedure

The study was publicised via employee mailing lists, social media (official Facebook and Twitter groups), and regular departmental mailings and publications. Study communications included a link to an online survey. Reminders were sent out weekly for six weeks between June and August 2020, with social media notifications posted daily in the final week before survey closure. Study posters were displayed in the wellbeing centres and on staff wellbeing noticeboards.

#### 2.4.1. Online Survey

Assumed consent was take from completion and submission of the survey. The platform used was Jisc Online Surveys (https://www.onlinesurveys.ac.uk), a platform selected due to compliance with UK data protection laws and the potential for access control, encryption and account security. Survey content was compiled by the research team and included questions about the participant’s age, gender, ethnicity, work status (e.g., employed, redeployed, volunteer, bank staff, healthcare student), their area of work, occupational group and level, whether they held any line manager responsibilities and whether they worked in a COVID-19 high risk area. The survey included a standardised measure of wellbeing, together with four single-item global measures of job stressfulness, job satisfaction, turnover intentions, presenteeism, and work engagement.

The measures included a 14-item measure of wellbeing (Warwick Edinburgh Mental Wellbeing Scale: WEMWBS [21]. The WEMWBS is a widely used scale to measure mental wellbeing in the general population. It is a short and psychometrically robust scale, with no ceiling effects in population samples [22]. The scale is scored by summing responses to each item answered on a 1 to 5 Likert scale. The minimum scale score is 14 and the maximum is 70, with higher scores indicating more positive wellbeing. Mean scores were calculated, and participants were classified as having high, average, or low mental wellbeing, using a cut-point of 40 or lower as indicative of poor mental wellbeing [23].

The survey included the following four single-item global measures. Job stressfulness was measured by the item: ‘In general, how stressful do you find your job?’ with responses on a 5 point scale ranging from 1 = ‘not at all stressful’ through to 5 = ‘extremely stressful’ [24]. Job satisfaction was measured by the item: ‘Taking everything into consideration, how do you feel about your job as a whole?’ with responses ranging from 1 = extremely dissatisfied through to 5 = extremely satisfied [25]. Turnover intentions were assessed using the item: ‘Are you considering leaving your job?’ (yes or no) [26]. Presenteeism was assessed using the item: ‘As far as you can recall, has it happened over the previous 12 months that you have gone to work despite feeling that you really should have taken sick leave due to your state of health?’ with responses options 1 = no, never, 2 = yes, once, 3 = yes, 2 to 5 times, 4 = yes, more than 5 times [27].

Employees were also asked to complete the dedication sub-scale of the 9-item Utrecht Work Engagement Scale (UWES-9, 3 items: DE2, DE3, DE4). Work engagement is considered to be the antipode of burnout. The scale has good internal consistency and test-retest reliability [28]. This measure required participants to report their level of agreement with the following statements: ‘I am enthusiastic about my job’, ‘My job inspires me’, ‘I am proud of the work I do’. Responses were on a 6-point scale ranging from 0 (never) to 6 (always/every day). The mean scale score of the UWES subscales is computed by adding the scores on the subscale and dividing the sum by the number of items of the subscale involved. Hence, the UWES dedication sub-scale yields a mean score that ranges between 0 and 6. Normative scores for this sub-scale (*n* = 9679; m = 3.88, s.d. = 1.38) have been classified as very low (<1.33), low (1.34–2.90), average (2.91–4.70), high (4.71–5.69), very high (≥5.70). In addition to means, scoring percentages may also be compared. In order to make this possible, the scores on the dedication sub-scale of the UWES were recorded in line with the scoring manual as follows: 1 = 0 to 0.99, (once a year or less), 2 = 1 to 1.99 (at least once a year), 3 = 2 to 2.99 (at least once a month), 4 = 3 to 3.99 (at least a couple of times a month), 5 = 4 to 4.99 (at least once a week), 6 = 5 to 6 (a couple of times per week or daily) [29]. Normative scoring distribution in percentages for the UWES-9 dedication sub-scale (*n* = 12,631) are 1.5 (1), 4.1 (2), 9.5 (3), 18.0 (4), 25.6 (5), 41.3 (6).

Twelve question items were included to investigate access to and use of the centres. Participants were asked whether they were aware of the wellbeing centres (yes; no), and whether they had accessed a centre (no; yes, once; yes, more than once). If they had not accessed a centre, they were asked to provide their main reason. Those that had visited a centre were asked to rate their overall level of satisfaction with the visit(s) on a scale of 1 to 10, where 1 = very dissatisfied and 10 = very satisfied. Participants that had accessed a centre were asked to confirm which they had visited (Site A, Site B or both), and their main reason for attending. Participants were asked whether they had talked to a buddy (no; yes, once; yes, more than once) and if so, to rate their overall level of satisfaction with their contact with the buddy on a scale of 1 to 10, where 1 = very dissatisfied and 10 = very satisfied. They were asked to provide the main benefits of accessing the centre(s), and any barriers or obstacles to accessing or using the centres. Finally, participants were invited to provide open-ended comments or suggestions for future provisions.

#### 2.4.2. Facility Monitoring

Attendance was recorded for 17 weeks between 6 April and 31 July 2020 (number of attendances, weekly, by site). Centre monitoring forms were introduced for an 11-week period from 18 May to 31 July 2020 (completed by new attenders only, excluding repeat visitors). Time of attendance was recorded on the forms to determine the most popular visiting times. Data collected included primary reason for attendance (e.g., quiet rest, social contact, access to resources, or conversation with buddy). Details on ethnicity were collected (e.g., white, mixed ethnicity, Asian or Asian/British, Black/African/Carribean/Black British, or other ethnic group) and occupational group of the visitor. Buddies recorded the number and nature of emotional help-seeking contacts; these were defined by the Buddies as ‘wobbles’ and referred to visitors actively seeking emotional support from the Buddies when distressed. Wobbles were categorised into ‘COVID-19 related’, ‘family concerns’, ‘personal matters’ (e.g., concerns for self, such as financial difficulties, relationship issues, work concerns), or ‘other’ (e.g., non-COVID guidance sought on behalf of others). Visitors were provided with an optional opportunity to provide a view or comment on the centres as feedback.

### 2.5. Data Analysis

Participant survey data and monitoring data were analysed using IBM SPSS Version 26.0 (IBM, Armonk, NY, USA). Descriptive statistics were computed by characteristics including: working in COVID-19 high or low risk area, centre access, buddy contact, and perceived benefits and barriers. The assumptions of normality of data were assessed by visualising histograms and P-P plots. Chi square test was applied to compare categorical variables e.g., characteristics versus COVID-19 high or low risk. We also used chi square test for comparison of characteristics according to centre access and buddy contact. Groups with small numbers were merged to conduct valid chi square tests. WEMWBS mean scores were roughly normally distributed, and were compared between participants with centre access/no access and COVID-19 high/low risk. For this comparison, we used independent samples t-test for two means, and one-way ANOVA for more than two means. Free text responses on the centre monitoring forms and the online survey were coded into broad themes.

## 3. Results

### 3.1. Facility Monitoring

**Figure 1:**
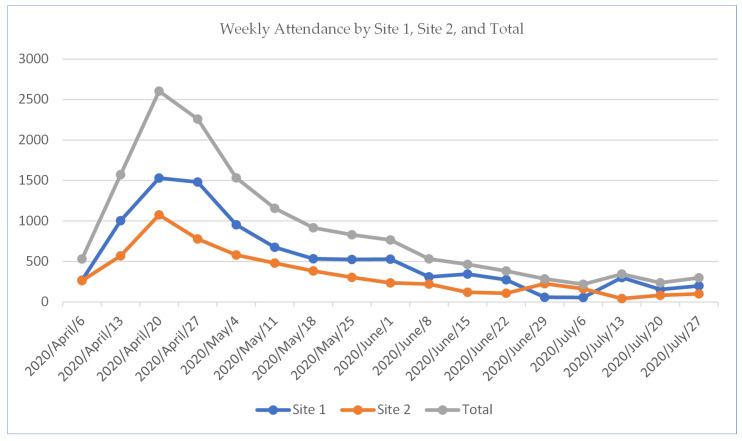
Weekly attendance at wellbeing centres.

Total number of attendances across 17 weeks was 14,934. Monthly attendance by site is shown in Table 1. Weekly attendance at Site 1 and Site 2 (for the 17-week period) is shown in Figure 1, visitor patterns were broadly comparable between sites and so attendance figures are reported for the wellbeing centres as a whole. There was a steady increase in attendance from 6 to 20 April (during the first peak of COVID-19 in the UK), with total weekly attendance rates ranging from 219 to 2605. The highest attendance across both sites in a single week was 20 April (*n* = 2605, Site 1 = 1530, Site 2; 1075). Overall, the highest attendance rates for the study period were through the month of April and early May (during and immediately after the first COVID-19 peak in the UK). The centres were more frequently utilised Monday to Friday and between the hours of 10:00–16:00. Forms were completed by wellbeing buddies to document ‘wobbles’ (staff actively help-seeking for emotional concerns). Within this record forms, there were 53 wobbles formally recorded (0.3% of overall attendances actively help-seeking for emotional concerns). Of these, 29 (52%) were directly related to concerns about COVID-19, 16 (28%) were related to concerns about family, 5 (9%) were related to personal matters, and 6 (11%) were other concerns. Examples of the reasons for emotional help-seeking and actions taken by the buddies are provided in Supplementary File 1. There were 375 feedback comments collated by the wellbeing buddies from new visitors. These comments related to the physical environment of the wellbeing centres as a calm and relaxing space (*n* = 101, 26.9%), general appreciation for the centres (*n* = 64, 17%), welcoming and supportive behaviour of the buddies (*n* = 46, 12.2%), and a desire for long-term maintenance of the centres (*n* = 25, 6.6%). The centres were described as ‘very tranquil’, ‘a supportive place for staff’ and ‘a great space to come and sit away from the stress of the hospital’. Buddies were described as ‘very friendly’, ‘approachable’ and ‘easy to talk to’. Visitors made reference to the benefits of these spaces with relation to their wellbeing, but also the prevention of sickness absenteeism: ‘It has kept me happy and at work during this stressful time’.

Total resource cost was £15,644, excluding administrative time for operational management and refreshments. Of this, centre set-up costs were £13,405. This included £7886.8 for fridges, microwaves, floor lamps, sensory lighting (borealis tubes), side tables, chairs and footstools, privacy screens, canvas pictures, aromatherapy diffusers, bluetooth speakers, eye masks and ear plugs. A further £5519 was allocated to the wellbeing centres for wipe clean chairs, sofas and equipment to ensure a minimum standard for the rooms. £2239 was spent on clinical psychology PFA training and supervisory support for 134 wellbeing buddies. This covered 10 group PFA sessions of 2 h each, delivered by 2 clinical psychologists (total: £2239; 20 h of PFA training; 40 h of trainer time), and 11 × 1 h supervision sessions spread throughout the intervention period (total: 13 h of supervision with 2 trainers at 11 sessions, and 1 trainer at 2 sessions).

### 3.2. Online Survey

#### 3.2.1. Survey: Participant Characteristics

There were 819 employees who completed the online survey. Of these, 308 (37.7%) reported that they were currently working in a COVID-19 high risk area (e.g., COVID-19 +ve ward, intensive care unit, emergency department, ward with COVID-19 patients, entrance meet and greet, staff or regular visitor to care or residential home, or other self-defined high risk area). The majority of those who self-defined as low risk were working from home. Participant characteristics are provided in Table 2. Respondents were broadly representative of employees at the participating hospital trust. Respondents were from diverse age groups, genders (88%F), and ethnic groups. Participants represented all divisions at the participating trust across a broad range of occupational groups. Over two-thirds of responses (67%) were from nurses, midwives, nursing and healthcare assistants and allied health professionals (AHPs) who were the most prevalent occupational groups in high risk areas. More than one-third of respondents declared that they had line manager responsibilities (37%).

#### 3.2.2. Survey: Wellbeing and Job Perceptions

Scores on the WEMWBS ranged from 14–70 (m = 46.16, s.d. = 9.46). Mean score was therefore marginally lower than WEMWBS Population Norms in Health Survey for England data 2011 (*n* = 7020, m = 51.61, s.d. = 8.71). Overall, participants were classified as having high (*n* = 68, 8.3%), average (*n* = 537, 65.6%), or low (*n* = 214, 26.1%) mental wellbeing. A comparison of WEMWBS mean scores by sample characteristics is shown in Supplementary File 2.

There were no statistically significant differences in wellbeing mean scores according to gender, ethnicity, work status, and COVID-19 high/low risk working area. However, there were significant differences in mean scores for WEMWBS with age (*p* = 0.02), occupational group (*p* = 0.002), level of seniority (*p* ≤ 0.001), and line manager responsibility (*p* = 0.002). Specifically, wellbeing scores were higher in older compared with younger participants (60+ years: m = 50.27, s.d. = 13.0; 21–30 years: m = 44.65, s.d. = 8.6). There was a significant difference in wellbeing according to level of seniority (*p* ≤ 0.001), with wellbeing mean scores highest at level 7 (senior roles) and for volunteers, and lowest at levels 1–2 (least senior roles). Comparison between groups showed statistically significant differences between levels 1–2 compared with level 7 (*p* = 0.001), levels 1–2 compared with volunteers (*p* = 0.001), levels 3–4 compared with level 7 (*p* = 0.01), and levels 3–4 compared with volunteers (*p* = 0.006). The lowest wellbeing scores were reported by ambulance workers and non-nursing clinical support workers, with the highest wellbeing reported by Trust grade/clinical fellows and staff from central/corporate functions. Statistically significant differences were found between central corporate x non nursing clinical support workers (*p* = 0.01), and medical/dental staff compared with non-nursing clinical support workers (*p* = 0.02). Those with line manager responsibilities were significantly more likely to report higher wellbeing (*p* = 0.002), and wellbeing scores were significant higher in those who had accessed the wellbeing centres compared with those who had not.

For the 819 participants, job stressfulness scores ranged from 1–5 (m = 2.79, s.d. = 0.87). High job stress was reported by 17.7% (*n* = 144) of respondents, and job dissatisfaction was reported by 18.1% (*n* = 148; range 1–5; m = 3.54, s.d. = 1.14), with 81.8% of respondents reporting job satisfaction (*n* = 669).

With regards turnover intentions, almost one-third of employees (*n* = 257, 31.6%) indicated that they had considered leaving their job. More than two-thirds (*n* = 560, 68.6%) of employees reported presenteeism (going into work when they should really have taken sick leave due to their health). Of those responding yes to this item, 74 employees (13.21%) had done this more than 5 times in the past 12 months. With regards engagement, almost three-quarters of employees (*n* = 606, 74.5%) reported that they were enthusiastic about their job (either often, very often or always). Nearly two-thirds of the sample (*n* = 514, 63.3%) felt that their job inspired them (either often, very often or always). The majority (*n* = 658, 80.6%) felt proud of the work they did (either often, very often or always).

Mean scores for the UWES dedication subscale (enthusiasm, inspiration and proud of work) were classified as high across the whole sample (compared with normative scores for this subscale provided in the manual: *n* = 9679; m = 3.88, s.d. = 1.38), but were significantly higher for those staff who were working in COVID-19 higher risk areas (m = 5.10, s.d. = 1.06) compared to staff working in COVID-19 lower risk areas (m = 4.83, s.d. = 1.20), and higher in those who had accessed the wellbeing centre (m = 5.02, s.d. = 1.14) compared to those staff who had not accessed a wellbeing centre (m = 4.83, s.d. = 1.15). Cronbach’s Alpha for the UWES subscale was 0.89 indicating good internal reliability (defined as 0.83–0.93 in Schaufeli and Bakker, 2004). Wellbeing and job perceptions by COVID-19 exposure are shown in Table 3. Participants working in COVID-19 higher risk areas reported a significantly higher level of job stressfulness than those who were working in lower risk areas. However, there were no differences in job satisfaction or turnover intentions between those working in higher or lower risk areas. Those working in higher risk areas reported greater work engagement on the dedication subscale of the UWES (enthusiasm for the job, inspired by the job, and proud of their work) compared with those in lower risk areas.

#### 3.2.3. Survey: Evaluation of the Wellbeing Centres

The vast majority of the respondents, (*n* = 767, 94%) were aware of the wellbeing centres. More than half of the respondents (*n* = 450, 55.2%) reported that they had accessed a centre. Of these, 448 participants reported which Sites they had visited: 261 had visited Site A, 140 had visited Site B, and 47 had visited both Sites A and B. Staff reported benefits (*n* = 450; 55.2% of the whole sample) and barriers to access (*n* = 365; 44.8% of the whole sample) as shown in Table 4. Additional reasons for non-attendance included being off work, on sick leave, opening times not being convenient, personal reasons (e.g., shyness) and staff believing the room should be prioritised for frontline workers.

Those that had not accessed a centre gave the following reasons: breaks were not long enough (*n* = 102, 27.8%), the centre was too far away from their place of work (*n* = 97, 26.4%), they were unable to take a break to attend (*n* = 77, 21%), they would prefer to take a break in a private rather than public space (*n* = 69, 18.8%), they had not felt the need (*n* = 60, 16.3%), they were working remotely/working from home (*n* = 45, 12.3%), they lacked awareness of the centres or whether non-clinical staff were able to attend (*n* = 38, 10.4%). there was not enough space or seating in the centre (*n* = 15, 4.1%), there was no buddy available (if accessing help was a reason for attendance) (*n* = 7, 1.9%). Fifty-one (13.9%) participants reported other reasons including the opening times were inappropriate and some of them thought that the centres should be used by staff who are working in COVID-19 areas.

Overall participant satisfaction ratings with the wellbeing centres ranged from 1–10 (m = 8.15, s.d. = 2.27). Participant’s main reasons for accessing a centre were: quiet rest (*n* = 287, 64.2%), social contact with peers (*n* = 184, 41.4%), to have a conversation with a buddy (*n* = 100, 22.6%), to get signposting or referral to other services (*n* = 33, 7.5%), or to access resources and information (*n* = 111, 25.2). A minority attended for other reasons, such as curiosity or accompanying a colleague.

Characteristics of participants who had accessed the centres are shown in Table 5. Of respondents who accessed the centres, 12.8% (of 811 providing details of their ethnicity) were from black or minority ethnic groups (BAME), which is lower than the proportion of BAME workers employed within the participating trust at the time of the study (35% of 18,011). The centres were most likely to be accessed by registered nurses or midwives, AHPs or nursing/healthcare assistants. They were least likely to be accessed by staff working in maintenance or general management, doctors in training or clinical fellows, and ambulance workers. Of those respondents who had accessed a centre, 83.3%, (*n* = 412) indicated that they would like to access one again.

Wellbeing and job perceptions were compared between those who had accessed the centres and those who had not (Table 3). There were no significant differences in perceived job stressfulness, job satisfaction, presenteeism or turnover intentions between those who did, or did not, access a centre. However, participants who had accessed a centre were more likely to report higher work engagement that those who did not access a centre. That is, mean scores for the UWES dedication subscale (enthusiasm, inspiration, and proud of work) in those who accessed a centre were higher than normative scores for this subscale (*n* = 9679; m = 3.88, s.d. = 1.38), and were significantly higher for those staff who accessed a wellbeing centre (m = 5.02, s.d. = 1.14) compared to staff who did not access a centre (m = 4.83, s.d. = 1.15).

There were 288 additional comments provided on the survey as free-text responses, which were categorised into 5 broad themes. The vast majority were expressions of appreciation for the centres (theme 1), comments related to the peaceful environment and positive impacts on wellbeing (theme 2). Overall, the comments suggested that the centres had positive impacts on staff wellbeing. There were many requests for the centres to be retained in the future (theme 3). Barriers (theme 4) included not feeling sanctioned to visit the centres by their managers or reports of centre opening times not being appropriate for the respondent. Suggested improvements to the provision (theme 5) included opening additional rooms and broadening the opening times.

## 4. Discussion

To our knowledge, this is the first study to evaluate the usage and impact of supported wellbeing centres implemented to mitigate the psychological impact of the COVID-19 pandemic on healthcare workers.

### 4.1. Centre Use and Access

The wellbeing centres on two hospital sites were very highly accessed during the first wave of COVID-19 in the UK. The centres were most heavily accessed during and immediately after the peak of the pandemic; usage declined over time in line with patterns of COVID-19 admissions but there remained a steady flow of visitors to study end. The centres were accessed by employees from diverse occupational groups, although the highest users were frontline workers (e.g., nurses, midwives, healthcare assistants and allied health professionals) who were the most prevalent occupational groups who self-defined as working in COVID-19 high risk areas, and lowest users were those in office-based jobs and manual workers. Those least likely to access the centres were maintenance staff, doctors in training, clinical fellows and ambulance workers. Ambulance workers are likely to have been primarily working away from site although it should be noted that this occupational group did not commonly access these rest areas on the main hospital sites. A lower attendance of office workers is likely to reflect the high numbers of staff who were remote working or shielding during this time, although feedback indicated that many staff in non-clinical roles were unaware that they were able to attend, felt they were not sanctioned to attend, or believed that clinical staff should be prioritised in a facility with limited space. Despite the clear need for high-quality rest areas, it is important to note the practical issues of identifying adequate spaces for rest areas in acute hospital sites. In this instance, conversion of two spaces into wellbeing centres was opportunistic and related to postponement of usual activities taking place in these areas. As usual services resume, space management and the identification of new sites for wellbeing centres becomes a significant challenge.

The most common reason for visiting a centre was quiet rest and recuperation, suggesting an ongoing need for time-out facilities and rest spaces for hospital workers. This is essential since work breaks may reduce the risk of burnout [30], and high-quality rest areas have been identified by healthcare workers as important in terms of their potential to positively influence staff, patient and facility outcomes [31]. It was notable that some employees reported that they were unable to take a break, or that breaks were not long enough to visit the centres due to their location. It seems likely that the provision of multiple rest spaces located closer to clinical areas would be more inclusive, although the issue of missed work breaks requires further consideration. The reasons for missed breaks are not known, although this could be related to the increased pressures on staff working through the pandemic or alternatively, may be related to ongoing issues with staffing and culture in healthcare services. These structural and job-related barriers to accessing well-being services need to be addressed. Further, the promotion of wellbeing initiatives should be coupled with awareness raising around protected work breaks, and the impacts of fatigue on well-being and care quality. Many of the centre users flagged that hydration was a key benefit of centre attendance. Advocating hydration is essential since dehydration has been found to be prevalent in frontline health and medical staff with negative consequences for cognitive function and performance [32,33]. Dehydration can arise when staff are working in warm environments, or they are focused on other things, missing their work breaks and therefore refreshments. Also, during the pandemic, there have been many anecdotal reports of healthcare workers skipping drinks in order not to waste personal protective equipment (PPE).

Other common reasons for access were for social contact and peer support, or to access wellbeing resources and signposting from a wellbeing buddy. Many of the employees that used the facilities had interacted with a wellbeing buddy during their visit. For some, this contact was for refreshments, social conversation, general signposting or advice. Others were emotionally distressed (albeit a small proportion of total visitors) and this was primarily related to the psychological impact of COVID-19 on themselves, colleagues or their families. In these instances, buddies were required to deliver psychological first aid (PFA). An important part of PFA is active listening which was the strategy most commonly used by the buddies. Examination of centre monitoring records indicated that buddies actively demonstrated an awareness of cultural preferences and needs and adopted a non-judgemental approach. They provided guidance around COVID-19 policies and engaged in promoting wellbeing and fostered active coping through encouraging access to support from friends, family, peers, supervisors or managers.

Staff accessed support from and were highly satisfied with the buddies. However, the service model of two buddies staffing two centres on a rolling rota was deemed to be unsustainable for the long-term. This was due to access to trained volunteers and resource implication for buddy training and ongoing support from clinical psychology services. However, there may be alternative approaches to achieving support for psychological wellbeing. Digital signposting to resources and support for UK health and care workers has been available since the early stages of the pandemic [7], and within such resources, social support is advocated as an important aspect of mitigating the psychological impact of COVID-19 [7]. Various other approaches have been utilised during the pandemic that are based around social support or personal contact. For example, individual peer buddy systems, e.g., ‘battle buddies’ [34], peer support groups using telehealth [35] or the use of social media [36]. An alternative would be to increase the proportion of staff who are trained in PFA at a local level, such as line managers or dedicated team members.

There was a significant financial and human resource cost in setting up and running the centres as well as the additional costs of clinical psychology services for the training and supervision of a large number of wellbeing buddies. As usual demands on service provider time resumed, alternative approaches to rest areas and psychological support are required. Although the resource investment was seen to be unsustainable, the investment made should be interpreted in the content of the costs of sickness absence due to mental health concerns. Mental health problems alone cost UK employers £45 bn each year (increase of 16% since 2017) due to absence costs, presentism and staff turnover, with an average return of £5 for every £1 invested [37]. The cost of poor mental health in the NHS equates to £1794–£2174 per employee per year [38]. In April 2020, the overall sickness absence rate in the UK was 6.2% in April 2020, and the most reported reason for sickness absence was mental health at 20.9% (anxiety, stress, depression or other psychiatric illnesses) [37].

### 4.2. Wellbeing and Job Perceptions

Wellbeing scores were higher in those who had accessed the wellbeing centres compared with those who had not. In the overall sample, over one quarter had low mental wellbeing on the WEMWBS and this was lower than that observed in general population samples. Wellbeing was lowest in younger staff and lower paid staff, and non-nursing clinical support workers and ambulance workers reported lower wellbeing than any other occupational group. This aligns with research conducted during the first peak of COVID-19 in the UK, which suggested that younger healthcare workers and people in lower paid roles have been disproportionally affected by the pandemic [39]. The negative wellbeing impacts on younger healthcare workers could be related to lower levels of experience in the job role, or the impacts of the pandemic on caregiving responsibilities [40]. NHS workforce research conducted by the UK Health Foundation has shown that staff in lower paid positions (staff earning less than £24,000 making up 40% of the NHS workforce [41] have poorer health and are more likely to have long-term health conditions than colleagues in higher paid posts [40]. Additionally, a Scottish Government report showed that NHS staff in lower paid roles have been found to have high levels of work-related stress, and are less likely to be aware of services to support their health and wellbeing [42]. Ambulance personnel have been found to be at particularly high risk for mental health concerns [43]. It should be noted that, in this context, ambulance workers are employed by the regional ambulance service rather than the participating hospital trust, although they were offered access to the trust wellbeing centres. It is clear that efforts should be made to deliver supportive interventions that include and support younger and lower paid staff (e.g., including clinical support workers). Moreover, further exploration may be needed to determine the wellbeing needs of ambulance workers.

Presenteeism is common in healthcare workers [27]. However, sickness presenteeism is of particular concern during the COVID-19 pandemic. Many individuals with COVID-19 may have only mild symptoms, and therefore there is a risk of healthcare workers inadvertently increasing local transmission through being present at work in ill-health [44]. It is possibly due to staff shortages and organisational culture setting norms against taking sick leave [45,46]. Another reason could be a sense of dedication to their work, which was demonstrated in our study by high levels of work engagement in these hospital staff. Turnover intentions in this sample were concerning, but within ranges identified in a national study of UK nurses prior to the COVID-19 pandemic (30–50%), where intentions to leave have been associated with demoralisation, failures in leadership, and perceived lack of support from managers which was seen to impact on nurses’ ability to provide safe, high quality care [47].

Although job stress was reported by many participants (as would be expected during a global pandemic), healthcare workers in this sample had higher job satisfaction (81%) than has been observed in other studies of public sector workers, although high levels of presenteeism (83%) were broadly comparable, e.g., social workers: 51% and 85% respectively, [48]. Work engagement was high in these hospital workers—particularly in those who worked in COVID-19 higher risk areas—with a high proportion reporting enthusiasm towards their job, being inspired by their job and feeling proud of the work they did. This could demonstrate the positive impact of working through a pandemic and is important since work engagement of NHS employees has been associated with a variety of individual and organisational outcome measures, including staff absenteeism and turnover, patient satisfaction and mortality, and safety measures, including infection rates [49].

### 4.3. Study Considerations

The participants represented a small proportion of a large workforce. However, responses were received from employees across diverse occupational groups, and the sample demographics were broadly comparable with the overall employee population. Figures for centre usage are based on the total number of visits. Since names were intentionally not recorded, it was not possible to determine the actual number of repeat visitors although estimates related to this were available from the survey.

Monitoring data were gathered by the buddies who were present in the rooms. This process was unavoidable since during the pandemic, there was a national COVID-19 lockdown and the research team were therefore remote working. The capacity of buddies to accurately capture attendance is likely to have been impacted by competing demands, and as such, attendance rates may be underestimated. The April spike is likely to reflect the public health significance of this month as the peak and immediate aftermath of the first wave of the COVID-19 pandemic in the UK. However, this was confounded by the availability of charitable donations for healthcare workers that were available for collection at the centres during this spike, and this will have significantly increased the number of visitors to the room at that time. Number of visitors may have been temporarily impacted at Site B by a period of access challenges due to a technical issue (faulty swipe card access) during weeks 1–3. The number of wobbles is highly likely to be underestimated due to the challenges of form filling by buddies during the provision of ‘in the moment’ emotional support during the peak of the pandemic.

### 4.4. Key Recommendations

We propose some key actions for healthcare employers, outlined in Table 6.

## 5. Conclusions

To our knowledge, this is the first study to evaluate the impact of COVID-19 staff wellbeing centres set up in an acute hospital setting to mitigate the psychological impact of COVID-19 on healthcare workers. The initiative was successful during the first wave of the pandemic since the centres were highly accessed and staff satisfaction ratings were high. Overall, job satisfaction and work engagement were prevalent among healthcare staff who are making a huge national contribution during a time of national crisis. However, the evaluation highlighted issues with employee presenteeism and turnover intentions which need to be addressed. Structural and job-related barriers hampered centre access for some staff, and the service model was resource-intensive meaning that alternative approaches are required for future sustainability.

## Figures and Tables

**Table 1:** Monthly wellbeing centre attendance by site during April–July 2020.

Site	April	May	June	July
Total	6967	4435	2431	1101
Site 1	4282	2687	1518	712
Site 2	2685	1748	913	389

**Table 2:** Participant Characteristics by COVID-19 risk area.

Characteristics (*n* = 819, 100%)	COVID-19 High Risk Exposure ^+^ (*n* = 308, 37.7%)	COVID-19 Low Risk Exposure ^++^ (*n* = 508, 62.3%)	Full Sample (*n* = 819, 100%)
Age (*n* = 817)			
16–20	2 (0.6)	5 (1)	7 (0.9)
21–30	69 (22.4)	74 (14.6)	144 (17.6)
31–40	69 (22.4)	109 (21.5)	178 (21.9)
41–50	88 (28.6)	146 (28.9)	236 (28.7)
51–60	67 (21.8)	155 (30.6)	222 (27.3)
60+	13 (4.2)	17 (3.4)	30 (3.7)
Gender (*n* = 819)			
Male	46 (14.9)	39 (7.7)	85 (10.4)
Female	259 (84.1)	459 (90.4)	721 (88)
Non-binary/gender fluid	1 (0.3)	2 (0.4)	3 (0.4)
Prefer not to disclose	2 (0.6)	8 (1.6)	10 (1.2)
Occupational group (*n* = 818)			
Registered Nurses/Midwives	140 (45.6)	155 (30.5)	296 (36.2)
Admin/clerical	24 (7.8)	123 (24.2)	147 (18)
Central/Corporate Functions	7 (2.3)	20 (3.9)	27 (3.3)
Medical & Dental	20 (6.5)	14 (2.8)	34 (4.2)
General Management	2 (0.7)	15 (3)	17 (2.1)
Ancillary/Maintenance	1 (0.3)	4 (0.8)	5 (0.6)
Nursing/Healthcare Assistants	32 (10.4)	51 (10)	84 (10.2)
Doctor in training	8 (2.6)	6 (1.2)	14 (1.7)
Ambulance	3 (1)	-	3 (0.4)
Trust grade/Clinical Fellow	3 (1)	1 (0.2)	4 (0.5)
Non nursing clinical support	5 (1.6)	14 (2.8)	19 (2.3)
AHP/Healthcare Scientists/Scientific & Technical	62 (20.2)	105 (20.7)	168 (20.5)
Job band ^1^ (*n* = 813)			
1–2	35 (11.5)	70 (13.8)	106 (13)
3–4	30 (9.9)	108 (21.3)	138 (17)
5–6	150 (49.3)	178 (35.2)	329 (40.5)
7	55 (18.1)	89 (17.6)	145 (17.8)
8A–D	8 (2.6)	39 (7.7)	47 (5.8)
9 and above	-	-	-
Other (e.g., volunteer)	26 (8.6)	22 (4.3)	48 (5.9)
Ethnicity (*n* = 815)			
White	261 (85.3)	446 (88.1)	710 (87.1)
Mixed Ethnicity	5 (1.6)	16 (3.2)	21 (2.6)
Black African/Caribbean/Black British	10 (3.3)	15 (3)	25 (3.1)
Asian/Asian British	25 (8.2)	16 (3.2)	41 (5)
Other ethnic groups	5 (1.6)	13 (2.6)	18 (2.2)
Line Manager Responsibilities (*n* = 811)			
Yes	123 (40.5)	176 (34.9)	300 (37)
No	181 (59.5)	328 (65.1)	511 (63)
Division (*n* = 817)			
Corporate	9 (2.9)	60 (11.8)	69 (8.5)
Estates & Facilities	3 (1)	10 (2)	13 (1.6)
Clinical Support	85 (27.7)	79 (15.6)	164 (20.1)
Family Health	22 (7.2)	82 (16.2)	104 (12.8)
Cancer and Specialties (CAS)	44 (14.3)	88 (17.4)	133 (16.2)
Medicine	84 (27.4)	66 (13)	151 (18.4)
Surgery	48 (15.6)	97 (19.1)	145 (17.8)
Treatment Centre	2 (0.7)	15 (3)	18 (2.1)
Other	10 (3.3)	10 (2)	20 (2.5)
Work status (*n* = 816)			
Redeployed	30 (9.8)	13 (2.6)	43 (5.3)
Employee of NUH	262 (85.3)	466 (92.1)	731 (89.5)
Bank staff	4 (1.3)	13 (2.6)	17 (2.1)
Student	3 (1)	3 (0.6)	6 (0.7)
Volunteer	3 (1)	5 (1)	8 (1)
Other	5 (1.6)	6 (1.2)	11 (1.4)

Note: ^+^ High-risk refers to working on COVID-19 +ve ward, intensive care unit, emergency care setting, care or residential home, entrance meet & greet, or other self-defined high-risk area (e.g., other ward with COVID-19 patients). ^++^ Low risk refers to home working or other self-defined low risk setting. ^1^ Further details/examples of jobs at each level available at: https://www.healthcareers.nhs.uk/working-health/working-nhs/nhs-pay-and-benefits/agenda-change-pay-rates.

**Table 3:** Wellbeing and job perceptions by COVID-19 exposure and Centre Access (*n* = 819).

Item	COVID-19 High Risk Exposure Mean (s.d.) or *n* (%)	COVID-19 Low Risk Exposure Mean (s.d.) or *n* (%)	Comparison (*p* ^+^)	Access Mean (s.d.) or *n* (%)	No Access Mean (s.d.) or *n* (%)	Comparison (*p* ^+^)
Mental Wellbeing						
WEMWBS ^‡^ Total Score	46.56 (10.07)	45.92 (9.05)	0.26	47.04 (9.49)	45.11 (9.35)	0.02 *
Job stressfulness	2.97 (0.90)	2.68 (0.83)	<0.001 ***	2.82 (0.87)	2.75 (0.87)	0.17
Job satisfaction	3.56 (1.14)	3.54 (1.14)	0.67	3.55 (1.12)	3.54 (1.16)	0.89
Intentions to leave			0.64			0.25
No	207 (67.6)	349 (69.2)		312 (38.27)	241 (29.58)	
Yes	99 (32.4)	155 (30.8)		133 (16.31)	123 (15.09)	
Presenteeism			0.53			0.28
no, never	101 (32.8)	154 (30.5)		133 (16.31)	122 (14.97)	
yes, once	75 (24.4)	167 (33.1)		139 (17.05)	104 (12.76)	
yes, 2 to 5 times	104 (33.8)	138 (27.3)		138 (16.92)	103 (12.64)	
yes, more than 5 times	28 (9.1)	46 (9.1)		37 (4.53)	36 (4.41)	
Work engagement (UWES) ^†^						
Enthusiastic about job	4.46 (1.30) ^^^	4.28 (1.36) ^^^	0.06	4.44 (1.29)	4.24 (1.38)	0.04 *
Job inspires me	4.21 (1.39) ^^^^	3.92 (1.48) ^^^^	0.007 **	4.18 (1.42)	3.85 (1.47)	0.001 ***
Proud of my work	4.94 (1.17) ^^^^	4.56 (1.30) ^^^	<0.001 ***	4.81 (1.22)	4.57 (1.31)	0.006 **
Total UWES	5.10 (1.06) ^^^^	4.83 (1.20) ^^^^	0.002 **	5.02 (1.14)	4.83 (1.15)	0.008

Note: ^‡^ Warwick-Edinburgh Mental Wellbeing Scale; ^†^ UWES: Utrecht Work Engagement Scale, dedication sub-scale. ^^^ average (2.91–4.70), ^^^^ high (4.71–5.69). * Significant at 0.05 alpha level; ** Significant at the 0.01 alpha level; *** Significant at the 0.001 alpha level. ^+^ Comparison between groups.

**Table 4:** Perceived benefits of wellbeing centres and barriers to access.

Item	Frequency (*n*)	Percent of Responses ^†^ (%)	Percent of Cases ^‡^ (%)
Benefits of access (*n* = 450)			
Time out/work break	360	17.2	82.6
Rest and Relaxation	313	15.0	71.8
More hydrated	239	11.4	54.8
Improved mental wellbeing/less stressed	230	11.0	52.8
Social contacts/Peer support	169	8.1	38.8
Access to charitable donations	146	7.0	33.5
Better work relationships	126	6.0	28.9
More positive outlook	115	5.5	26.4
Chance to eat	89	4.3	20.4
Getting personal health or wellbeing advice	65	3.1	14.9
Better patient care	64	3.1	14.7
Changes to work activities	50	2.4	11.5
Signposted to other services	47	2.2	10.8
Getting job-related information or advice	41	2.0	9.4
Other benefit	36	1.7	8.3
Barriers to access (*n* = 365)			
Break not long enough	102	18.2	27.8
Room too far away	97	17.3	26.4
Unable to take a break	77	13.7	21.0
Prefer to take a break in private	69	12.3	18.8
Not felt the need	60	10.7	16.3
Remote working/working from home	45	8.0	12.3
Lack of awareness of the centres	38	6.7	10.4
Not enough space/seating	15	2.7	4.1
No Wellbeing Buddy available	7	1.2	1.9
Other barriers	51	9.1	13.9

^†^ Percentages of items in total selection. ^‡^ Percentages of participants in each total sample (450 who accessed a centre, 365 who did not access a centre).

**Table 5:** Participant characteristics by centre use and buddy contact.

Characteristics	Access (*n* = 450, 55.2%)	No Access (*n* = 365, 44.8%)	Total *n* = 819 (100%)	Comparison (*p* *)	Buddy Contact (*n* = 260, 57.7%)	No buddy Contact (*n* = 190, 42.3%)	Total (*n* = 450, 100%)	Comparison (*p* *)
Age (*n* = 813)				<0.008 **				0.98
16–20	4 (0.9)	3 (0.8)	7 (0.9)		2 (0.8)	2 (1.1)	4 (0.9)	
21–30	97 (21.6)	47 (12.9)	144 (17.7)		57 (22.1)	40 (21.1)	97 (21.7)	
31–40	105 (23.4)	72 (19.8)	177 (21.8)		60 (23.3)	45 (23.7)	105 (23.4)	
41–50	115 (25.6)	119 (32.7)	234 (28.8)		67 (26)	48 (25.3)	115 (25.7)	
51–60	111 (24.7)	110 (30.2)	221 (27.2)		61 (23.6)	49 (25.8)	110 (24.6)	
60+	17 (3.8)	13 (3.6)	30 (3.7)		11 (4.3)	6 (3.2)	17 (3.8)	
Gender (*n* = 815) ^+^				0.07				0.12
Male	37 (8.2)	47 (12.9)	84 (10.3)		27 (10.4)	10 (5.3)	37 (8.2)	
Female	407 (90.4)	311 (85.2)	718 (88.1)		228 (88)	178 (93.7)	406 (90.4)	
Non-binary/Gender fluid	2 (0.4)	1 (0.3)	3 (0.4)		2 (0.8)	0 (0)	2 (0.4)	
Prefer not to disclose	4 (0.9)	6 (1.6)	10 (1.2)		2 (0.8)	2 (0.8)	4 (0.9)	
Occupational group (*n* = 814) ^+^				<0.001 ***				0.39
Registered Nurses/Midwives	178 (39.6)	115 (31.6)	293 (36)		108 (41.7)	70 (36.8)	178 (39.6)	
Admin/clerical	56 (12.4)	90 (24.7)	146 (17.9)		24 (9.3)	31 (16.3)	55 (12.2)	
Central/Corporate Functions	15 (3.3)	12 (3.3)	27 (3.3)		9 (3.5)	6 (3.2)	15 (3.3)	
Medical & Dental	16 (3.6)	18 (4.9)	34 (4.2)		7 (2.7)	9 (4.7)	16 (3.6)	
General Management	6 (1.3)	11 (3)	17 (2.1)		5 (1.9)	1 (0.5)	6 (1.3)	
Ancillary/Maintenance	3 (0.7)	2 (0.5)	5 (0.6)		2 (0.8)	1 (0.5)	3 (0.7)	
Nursing/Healthcare Assistants	53 (11.8)	31 (8.5)	84 (10.3)		29 (11.2)	24 (12.6)	53 (11.8)	
Doctor in training	10 (2.2)	4 (1.1)	14 (1.7)		5 (2.6)	5 (2.6)	10 (2.2)	
Ambulance	-	3 (0.8)	3 (0.4)		-	-	-	
Trust grade/Clinical Fellow	3 (0.7)	1 (0.3)	4 (0.5)		2 (0.8)	1 (0.5)	3 (0.7)	
Non nursing clinical support	12 (2.7)	7 (1.9)	19 (2.3)		7 (2.7)	5 (2.6)	12 (2.7)	
AHP/healthcare scientists/scientific & technical	98 (21.8)	70 (19.2)	168 (20.60)		61 (23.6)	37 (19.5)	98 (21.8)	
Job band ^1^ (*n* = 809)				0.08				0.08
1–2	68 (15.3)	38 (10.4)	106 (13.1)		30 (11.7)	38 (20.2)	68 (15.3)	
3–4	66 (14.8)	72 (19.8)	138 (17.1)		41 (16)	24 (12.8)	65 (14.6)	
5–6	189 (42.5)	137 (37.6)	326 (40.3)		112 (43.8)	77 (41)	189 (42.6)	
7	75 (16.9)	69 (19)	144 (17.8)		48 (18.8)	27 (14.4)	75 (16.9)	
8A–D	22 (4.9)	25 (6.9)	47 (5.8)		14 (5.5)	8 (4.3)	22 (5)	
9 and above	-	-	-		-	-	-	
Other (e.g., volunteer)	25 (5.6)	23 (6.3)	48 (5.9)		11 (4.3)	14 (7.4)	25 (5.6)	
Ethnicity (*n* = 811)				0.65				<0.007 **
White	387 (86.4)	320 (88.2)	707 (87.2)		215 (83.3)	172 (91)	387 (86.6)	
Mixed Ethnicity	15 (3.3)	6 (1.7)	21 (2.6)		14 (5.4)	1 (0.5)	15 (3.4)	
Black African/Caribbean/Black British	14 (3.1)	11 (3)	25 (3.1)		11 (4.3)	2 (1.1)	13 (2.9)	
Asian/Asian British	22 (4.9)	19 (5.2)	41 (5.1)		14 (5.4)	8 (4.2)	22 (4.9)	
Other ethnic groups	10 (2.2)	7 (1.9)	17 (2.1)		4 (1.6)	6 (3.2)	10 (2.2)	
Line Manager Responsibilities (*n* = 807)				0.72				0.74
Yes	163 (36.6)	137 (37.8)	300 (37.2)		92 (36.1)	71 (37.6)	163 (36.30)	
No	282 (63.4)	225 (62.2)	507 (62.8)		163 (63.9)	118 (62.4)	281 (62.58)	
Division (*n* = 813)				<0.001 ***				0.59
Corporate	28 (6.2)	41 (11.3)	69 (8.5)		18 (6.9)	10 (5.3)	28 (6.3)	
Estates & Facilities	10 (2.2)	3 (0.8)	13 (1.6)		7 (2.7)	3 (1.6)	10 (2.2)	
Clinical Support	92 (20.5)	71 (19.5)	163 (20)		46 (17.8)	46 (24.3)	92 (20.5)	
Family Health	43 (9.6)	61 (16.8)	104 (12.8)		28 (10.8)	15 (7.9)	43 (9.6)	
Cancer and Specialities (CAS)	54 (12)	78 (21.4)	132 (16.2)		33 (12.7)	21 (11.1)	54 (12.1)	
Medicine	103 (22.9)	47 (12.9)	150 (18.5)		55 (21.2)	48 (25.4)	103 (23)	
Surgery	101 (22.5)	43 (11.8)	144 (17.7)		63 (24.3)	38 (20.1)	101 (22.5)	
Treatment Centre	8 (1.8)	10 (2.7)	18 (2.2)		4 (1.5)	3 (1.6)	7 (1.6)	
Other	10 (2.2)	10 (2.7)	20 (2.5)		5 (1.9)	5 (2.6)	10 (2.2)	
Work status (*n* = 812) ^+^				0.13				0.22
Redeployed	29 (6.5)	14 (3.9)	43 (5.3)		21 (8.1)	8 (4.2)	29 (6.5)	
Employee of NUH	392 (87.3)	335 (92.3)	727 (89.5)		222 (86)	169 (88.9)	391 (87.3)	
Bank staff	12 (2.7)	5 (1.4)	17 (2.1)		8 (3.1)	4 (2.1)	12 (2.7)	
Student	4 (0.9)	2 (0.6)	6 (0.7)		2 (0.8)	2 (1.1)	4 (0.9)	
Volunteer	6 (1.3)	2 (0.6)	8 (1)		4 (1.6)	2 (1.1)	6 (1.3)	
Other	6 (1.3)	5 (1.4)	11 (1.4)		1 (0.4)	5 (2.6)	6 (1.3)	

* Significant at the 0.05 alpha level; ** Significant at the 0.01 alpha level; *** Significant at the 0.001 alpha level. ^+^ In order to conduct a valid chi square test, groups with small sample sizes were merged: student, volunteer and other in ‘work status’ group, ancillary/maintenance, ambulance and trust grade/clinical fellow in ‘occupational group’, and non-binary/gender fluid and prefer to not disclose in ‘gender group’.

**Table 6:** Recommended actions for healthcare organisations.

Provision of Time-Out/Rest Spaces
Offer multiple dedicated wellbeing areas intended solely for rest and recuperation. Ideally, these should be proximate to, but separate from, clinical areas and should not be dual purpose.
**Recognise the needs of specific groups**
Target promotion of wellbeing initiatives to staff groups that may have particular wellbeing concerns or challenges with access to support (e.g., BAME staff, staff in lower paid roles, staff who are not based in central sites or have community-focused roles).
**Top-level advocacy and visibility**
Embrace a culture of wellbeing through top-down promotion and advocacy of wellbeing and visibility of leaders.
Raise awareness of the impact of COVID-19 on mental health and the risks to healthcare workers of stress, burnout and PTSD.
Ensure policy is in place around protected work breaks and undertake monitoring and evaluation of how policy is being implemented.
**Line manager training and support**
Raise awareness of presenteeism and the risks to potential healthcare-associated COVID-19 transmission, staff mental wellbeing and staff morale.
Provide line manager training and support to increase awareness of wellbeing policy and the importance of work breaks with relation to fatigue, dehydration and physical or mental ill-health.
Train line managers or dedicated team members in psychological first aid and signposting
Build psychologically safe work environments that allow workers to speak up when they have high stress, low wellbeing or have physical or mental ill-health (advocating compassionate leadership, civility and recognition of ‘It’s OK to not be OK’).
**Multiple support options**
Provide employees with access to group and individual counselling, pastoral and spiritual care, online platforms, psychological hotlines, peer support networks and stress-management tools
Ensure there is local support for employees in distress, e.g., dedicated wellbeing support workers, coaches or line managers trained in psychological first aid and signposting.
**Make retention a key strategic imperative**
Give reward and recognition for excellence and engagement
Foster teamwork and collaboration
Demonstrate a clear growth path—invest in education and continual learning

## Supplementary Materials

The following are available online at https://www.mdpi.com/1660-4601/17/24/9401/s1, File 1: Examples of emotional help-seeking and actions taken; File 2: Total wellbeing scores by sample characteristics

## References

[1] NHS (2018). Staff Health & Wellbeing: CQUIN 2017–19 Indicator 1 Implementation Support.

[2] Blake H., Zhou D., Batt M.E. (2013). Five-year workplace wellness intervention in the NHS. Perspect. Public Health.

[3] Adams J.G., Walls R.M. (2020). Supporting the health care workforce during the COVID-19 global epidemic. JAMA.

[4] Mitchell G. Third of Nurses Experiencing ‘Severe’ Mental Health Issues Due to Covid-19. https://www.nursingtimes.net/news/coronavirus/uk-nurses-feel-unprepared-for-COVID-19-peak-finds-new-survey-21-04-2020/.

[5] Gilroy R. Nurses Report Emerging Signs of Mental Health Strain DURING Covid-19 Peak. Nursing Times. https://www.nursingtimes.net/news/workforce/nurses-report-emerging-signs-of-mental-health-strain-during-covid-19-peak-21-05-2020/.

[6] Blake H., Bermingham F. (2020). Psychological Wellbeing for Healthcare Workers: Mitigating the Impact of COVID-19.

[7] Blake H., Bermingham F., Johnson G., Tabner A. (2020). Mitigating the psychological impact of COVID-19 on healthcare workers: A digital learning package. IJERPH.

[8] Malacrida C. (2005). Discipline and dehumanization in a total institution: Institutional survivors’ descriptions of Time-Out Rooms. Disabil. Soc..

[9] Hall L.H., Johnson J., Watt I., Tsipa A., O’Connor D.B. (2016). Healthcare staff wellbeing, burnout, and patient safety: A systematic review. PLoS ONE.

[10] Boehm H., Morast S. (2009). Quiet time. AJN Am. J. Nurs..

[11] Jones N. (2019). Tune-in and time-out: Toward surgeon-led prevention of “never” events. J. Patient Saf..

[12] Papadakis M., Meiwandi A., Grzybowski A. (2019). The WHO safer surgery checklist time out procedure revisited: Strategies to optimise compliance and safety. Int. J. Surg..

[13] Kisely S., Warren N., McMahon L., Dalais C., Henry I., Siskind D. (2020). Occurrence, prevention, and management of the psychological effects of emerging virus outbreaks on healthcare workers: Rapid review and meta-analysis. BMJ.

[14] Ford S. Trusts Setting Up ‘Wobble Rooms’ to Give Nursing Staff Some Covid-19 Respite. https://www.nursingtimes.net/news/workforce/trusts-setting-up-wobble-rooms-to-give-nursing-staff-with-COVID-19-respite-07-05-2020/.

[15] Bates J. Why the Wobble Room Is a Necessary Sanity Saver. https://rcni.com/nursing-standard/opinion/comment/why-wobble-room-a-necessary-sanity-saver-161236.

[16] Cummings K.S., Grandfield S.A., Coldwell C.M. (2010). Caring with comfort rooms. J. Psychosoc. Nurs. Ment. Health Serv..

[17] Hedlund Lindberg M., Samuelsson M., Perseius K.-I., Björkdahl A. (2019). The experiences of patients in using sensory rooms in psychiatric inpatient care. Int. J. Ment. Health Nurs..

[18] Rimmer A., Chatfield C. (2020). What organisations around the world are doing to help improve doctors’ wellbeing. BMJ.

[19] Blake H. (2020). Psychological Wellbeing of Nurses and Midwives During the COVID-19 Pandemic. Florence Nightingale Found..

[20] Committee I.-A.S. (2006). IASC Guidelines on Mental Health and Psychosocial Support in Emergency Settings.

[21] Stewart-Brown S., Janmohamed K. (2008). Warwick-Edinburgh Mental Well-Being Scale. User Guide. Version 1.

[22] Tennant R., Hiller L., Fishwick R., Platt S., Joseph S., Weich S., Parkinson J., Secker J., Stewart-Brown S. (2007). The Warwick-Edinburgh mental well-being scale (WEMWBS): Development and UK validation. Health Qual. Life Outcomes.

[23] Collect, Score, Analyse and Interpret WEMWBS. https://warwick.ac.uk/fac/sci/med/research/platform/wemwbs/using/howto/.

[24] Houdmont J., Jachens L., Randall R., Hopson S., Nuttall S., Pamia S. (2019). What does a single-item measure of job stressfulness assess?. Int. J. Environ. Res. Public Health.

[25] Dolbier C.L., Webster J.A., McCalister K.T., Mallon M.W., Steinhardt M.A. (2005). Reliability and validity of a single-item measure of job satisfaction. Am. J. Health Promot..

[26] Ryan S.V., Nathaniel P., Pendergast L.L., Saeki E., Segool N., Schwing S. (2017). Leaving the teaching profession: The role of teacher stress and educational accountability policies on turnover intent. Teach. Teacher Educ..

[27] Aronsson G. (2000). Sick but yet at work. An empirical study of sickness presenteeism. J. Epidemiol. Commun. Health.

[28] Schaufeli W.B., Bakker A.B., Salanova M. (2006). The Measurement of Work Engagement With a Short Questionnaire: A Cross-National Study. Educ. Psychol. Measur..

[29] Schaufeli W.B., Bakker A.B. (2004). Job demands, job resources, and their relationship with burnout and engagement: A multi-sample study. J. Organiz. Behav..

[30] Cordoza M., Ulrich R.S., Manulik B.J., Gardiner S.K., Fitzpatrick P.S., Hazen T.M., Mirka A., Perkins R.S. (2018). Impact of nurses taking daily work breaks in a hospital garden on burnout. Am. J. Crit. Care.

[31] Nejati A., Rodiek S., Shepley M. (2016). The implications of high-quality staff break areas for nurses’ health, performance, job satisfaction and retention. J. Nurs. Manag..

[32] El-Sharkawy A.M., Bragg D., Watson P., Neal K., Sahota O., Maughan R.J., Lobo D.N. (2016). Hydration amongst nurses and doctors on-call (the HANDS on prospective cohort study). Clin. Nutr..

[33] Parry D., Oeppen R.S., Gass H., Brennan P.A. (2017). Impact of hydration and nutrition on personal performance in the clinical workplace. Br. J. Oral Maxillofac. Surg..

[34] Albott C.S., Wozniak J.R., McGlinch B.P., Wall M.H., Gold B.S., Vinogradov S. (2020). Battle Buddies: Rapid deployment of a psychological resilience intervention for health care workers during the COVID-19 Pandemic. Anesth. Analg..

[35] Viswanathan R., Myers M.F., Fanous A.H. (2020). Support groups and individual mental health care via video conferencing for frontline clinicians during the COVID-19 pandemic. Psychosomatics.

[36] Cheng P., Xia G., Pang P., Wu B., Jiang W., Li Y.-T., Wang M., Ling Q., Chang X., Wang J. (2020). COVID-19 epidemic peer support and crisis intervention via social media. Commun. Ment. Health J..

[37] Deloitte (2020). Mental Health and Employers: Refreshing the Case for Investment. https://www2.deloitte.com/content/dam/Deloitte/uk/Documents/consultancy/deloitte-uk-mental-health-and-employers.pdf.

[38] Health Education England (2019). NHS Staff and Learner’s Mental Wellbeing Commission. https://www.hee.nhs.uk/sites/default/files/documents/NHS%20%28HEE%29%20-%20Mental%20Wellbeing%20Commission%20Report.pdf.

[39] Thomas C., Quilter-Pinner H., Research IfPP (2020). Care Fit for Carers: Ensuring the Safety and Welfare of NHS and Social Care Workers During and After Covid-19. Institute for Public Policy Research. www.ippr.org/files/2020-04/care-fit-for-carers-april20.pdf.

[40] Boisanger J. (2020). Valuing the Health and Wellbeing of Lower Paid NHS Staff UK. https://www.health.org.uk/news-and-comment/blogs/valuing-the-health-and-wellbeing-of-lower-paid-nhs-staff.

[41] UNISON Annual Report 2014/15. https://www.unison.org.uk/content/uploads/2015/04/On-line-Catalogue23144.pdf.

[42] Glasgow N.G. (2012). A Health Profile of the NHS Greater Glasgow and Clyde Workforce: With an Exploration of Health Issues of Lower Paid Staff.

[43] Petrie K., Milligan-Saville J., Gayed A., Deady M., Phelps A., Dell L., Forbes D., Bryant R.A., Calvo R.A., Glozier N. (2018). Prevalence of PTSD and common mental disorders amongst ambulance personnel: A systematic review and meta-analysis. Soc. Psychiatr. Psychiatr. Epidemiol..

[44] Wee L.E., Sim X.Y.J., Conceicao E.P., Aung M.K., Goh J.Q., Yeo D.W.T., Gan W.H., Chua Y.Y., Wijaya L., Tan T.T. (2020). Containment of COVID-19 cases among healthcare workers: The role of surveillance, early detection, and outbreak management. Infect. Control Hosp. Epidemiol..

[45] McKevitt C., Morgan M., Dundas R., Holland W.W. (1997). Sickness absence and ‘working through’ illness: A comparison of two professional groups. J. Public Health.

[46] Dew K. (2011). Pressure to Work through Periods of Short Term Sickness.

[47] Senek M., Robertson S., Ryan T., King R., Wood E., Taylor B., Tod A. (2020). Determinants of nurse job dissatisfaction—Findings from a cross-sectional survey analysis in the UK. BMC Nurs..

[48] Ravalier J.M. (2018). The influence of work engagement in social workers in England. Occupat. Med..

[49] West M., Dawson J. (2012). Employee Engagement and NHS Performance.

